# Effect of admission blood glucose on early mortality in patients with pontine hemorrhage

**DOI:** 10.1038/s41598-025-09659-8

**Published:** 2025-07-21

**Authors:** Junqing Ma, Junhong Hao, Lan Xu, Xiaowei Miao, Yanquan Hou, Zujun Song

**Affiliations:** 1 Emergency Department, The Eighth Medical Center of Chinese PLA General Hospital, Beijing, China; 2 Geriatrics Department, Xi’an Qinhuang Hospital, Xi’an, Shaanxi China; 3 Department of Medical Record, The First Hospital of Handan City, Handan, Hebei China; 4 Department of Medical Record, The Central Hospital of Handan City, Handan, Hebei China; 5 Second Pediatric Department, Affiliated Hospital of Hebei University of Engineering, Handan, Hebei China; 6 Emergency and Critical Care Hospital, Xi’an People’s Hospital (Xi’an Fourth Hospital), Xi’an, Shaanxi China

**Keywords:** Pontine hemorrhage, Blood glucose, Mortality, Prognosis, Stroke, Stroke

## Abstract

Elevated admission blood glucose (ABG) is increasingly recognized as a prognostic marker in intracerebral hemorrhage, but its specific role in pontine hemorrhage (PH) remains underexplored. This retrospective study analyzed data from 247 PH patients admitted to two tertiary hospitals between 2012 and 2016 to evaluate the association between ABG and 30-day mortality. Receiver operating characteristic analysis identified 8.69 mmol/L as the optimal hyperglycemia threshold predictive of mortality. Survival analysis revealed significantly lower 30-day survival rates in hyperglycemic patients compared to non-hyperglycemic patients (*P* = 0.0017). In multivariate regression models, ABG (per 1 mmol/L increase) independently increased mortality risk, with an adjusted odds ratio (aOR) of 1.295 (*P* = 0.0097) in the overall cohort and an aOR of 1.266 (*P* = 0.0210) in the nondiabetic patients. Hyperglycemia was significantly associated with increased mortality compared to non-hyperglycemia, with an aOR of 3.641 (*P* = 0.0024) in the overall cohort and an aOR of 3.492 (*P* = 0.0035) in the nondiabetic subgroup. Notably, no significant association was observed in diabetic patients (*P* > 0.05). These findings suggest that elevated ABG serves as an independent predictor of 30-day mortality following PH, particularly in nondiabetic populations, and may facilitate early risk stratification.

## Introduction

Pontine hemorrhage (PH), accounting for only 5–10% of all intracerebral hemorrhage (ICH) cases, carries a notoriously poor prognosis^1–4^. Identifying prognostic factors and implementing effective interventions are therefore of critical importance. Numerous studies have shown that elevated admission blood glucose (ABG) is associated with poor outcomes in patients with ICH^5–7^. However, most of these studies did not stratify patients based on the anatomical location of the hemorrhage. Because the proportion of PH to all ICH cases is relatively small, it has been unclear whether the conclusion from the overall ICH patients can be applied to PH. To the best of our knowledge, rarely has there been research on the relationship between ABG and prognosis specifically focused on PH. In only a few studies^8–11^the results were conflicting because of the lack of effective adjustment for confounders or the insufficient sample sizes. Therefore, the present study aims to assess the effect of ABG on early mortality in PH patients, while accounting for potential confounding factors, using a relatively large patient cohort.

## Methods

### Study population

We retrospectively reviewed the medical records of consecutive patients with PH who were admitted to the First Hospital and the Central Hospital of Handan City, two tertiary hospitals in northern China, between June 2012 and June 2016. Patients with acute PH confirmed by clinical assessment and either computed tomography or magnetic resonance imaging, those admitted to the hospital within 24 h of symptom onset, and individuals aged over 18 years were included in this analysis.

**Fig. 1:**
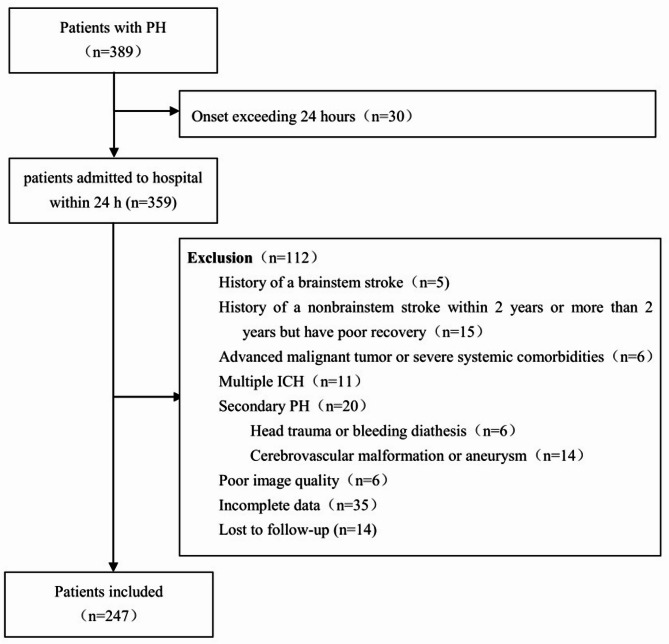
Flowchart of study selection.

The following patients were excluded from the analysis: (1) those with PH secondary to tumors, head trauma, bleeding diathesis, cerebrovascular malformation, aneurysm, or hemorrhagic infarction; (2) patients with multiple ICH; (3) individuals with a history of non-brainstem stroke within the past two years, or more than two years with poor recovery (Glasgow Outcome Scale score ≤ 4); (4) patients with any history of brainstem stroke; (5) cases involving advanced malignancies or severe systemic comorbidities; and (6) those with poor image quality, incomplete data or lost to follow-up (Fig. 1).

All methods were performed in accordance with the relevant guidelines and regulations. The study was approved by the medical ethics committee of the First Hospital of Handan City. As a retrospective review of existing data, specific informed consent was not obtained from the patients. However, informed consent was obtained from all patients on admission for all treatments. All data were delinked from individual identifying information and were analyzed anonymously. Waiver of the requirement for informed consent was obtained under the auspices of the medical ethics committee.

### Management

All patients were cared for in medical, surgical, or emergency intensive care units until they were considered stable enough to be moved to a general ward. The conservative treatment adopted, according to clinical routine, included controlling blood pressure (BP, early goal of maintaining systolic BP below 160 mmHg), regulating blood glucose (early below 10 mmol/L), reducing intracranial pressure (when computed tomography indicated a mass effect or when clinical symptoms showed an increased intracranial pressure), and systematically supporting treatment. Surgical evacuation of hematoma or continuous ventricular drainage was taken according to clinical indications and the discretion of patients or family members.

### Data collection and variable definition

Collected demographics and medical history included sex, age, and histories of hypertension and diabetes mellitus. Definitions of hypertension and diabetes mellitus were as described in previous publications^12,13^. The following clinical and laboratory data were collected on admission: body temperature, heart rate, abnormal respiration, BP, pupillary light reflex, Glasgow Coma Scale (GCS) score, hemoglobin, white blood cell, platelet, serum potassium, serum sodium, alanine aminotransferase, creatinine, and ABG. Abnormal respiration was defined as less than 12 or more than 24 breaths per minute or a requirement for mechanical ventilation. For patients who had undergone intubation, GCS verbal score was assigned 1 point for data analysis because of patients’ sinking into a coma on admission. Pupillary light reflex was qualitatively recorded as absent (lack of reactivity) or present (reactivity) using a traditional penlight. When bilateral pupillary light reflexs were not consistent, we recorded the more severe side.

All patients routinely underwent a computed tomography scan before admission following PH. The following radiological indicators were collected: volume and location of hematoma, vertical or intraventricular extension of hemorrhage, and hydrocephalus. The volume was measured using the ellipsoid formula (4/3π × A × B × C) where A, B, and C represent the respective radii (cm) in three-dimensional images^14^. Vertical extension of hemorrhage was divided into pons only and rostrocaudal extension (extension to midbrain, thalamus, or medulla oblongata). All computed tomography images were reviewed by a neuroradiologist blinded to clinical data.

### Measurements of outcome

Patients were classified into a survival group and a death group based on their 30-day outcomes. Information on the outcomes was obtained from medical records, family members via phone interviews, or direct examinations at the outpatient department after discharge.

### Statistical analysis

Continuous variables are expressed as mean (standard deviation) or median (interquartile range), as appropriate, while categorical variables are expressed as percentages. Receiver operating characteristic analysis was performed to determine an optimal cut-off value of critical hyperglycemia for 30-day mortality. Patients were divided into two groups based on the cut-off value: hyperglycemia and nonhyperglycemia groups. Kaplan–Meier survival curve analysis was performed to estimate survival rate for the two different ABG groups, and log-rank test was used to analyze the significance of difference between the two groups. A generalized additive model was used to explore potential linear association of ABG with mortality. Univariate analysis was performed to examine the effect of each potential confounder on mortality, and multiple logistic regression analysis was performed to examine the independent effect of ABG on mortality. In multiple regression analysis, selected confounders included variables biologically associated with 30-day mortality after PH, those statistically associated with mortality in univariate analysis (*P* < 0.10), or those that regression coefficient of ABG changed more than 10% when they were removed from full model (including all potential confounders) or entered in basic model (only ABG)^15^. However, multiple regression analysis did not include the variables whose variance inflation factor was 5 or higher. All analyses were performed using Empower (R) (www.empowerstats.com, X&Y solutions, Inc., Boston, MA) and R (http://www.R-project.org).

## Results

### Baseline characteristics of patients with PH

**Fig. 2:**
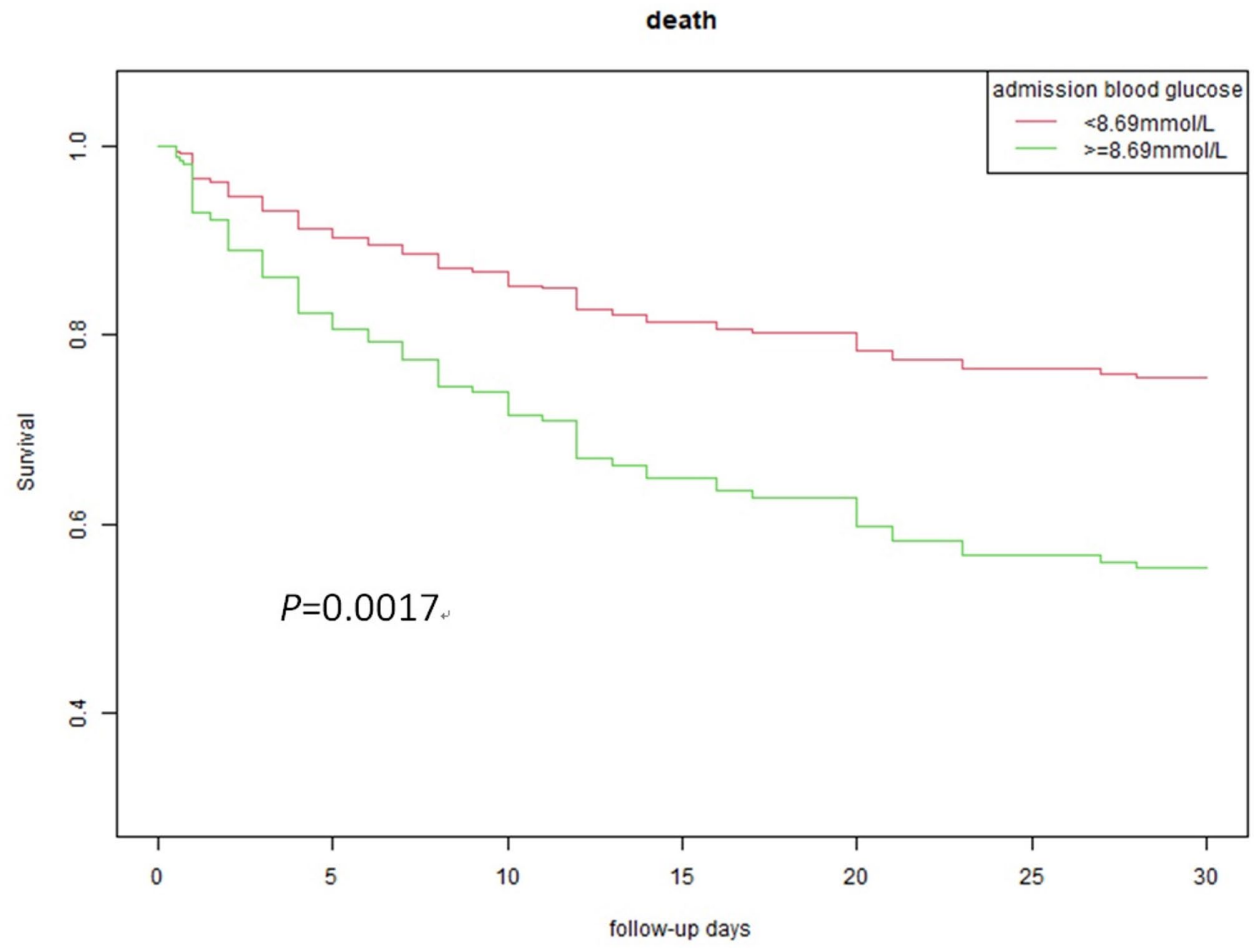
Survival at 30-day after pontine hemorrhage according to admission blood glucose. Adjusted for age, hypertension, diabetes, body temperature, heart rate, systolic BP, abnormal respiration, GCS score, pupillary light reflex, hemoglobin, white blood cell, creatinine, hemorrhagic volume, rostrocaudal and intraventricular extension, hydrocephalus.

A total of 247 patients were identified. 172 (69.6%) patients were men and 75 (30.4%) were women; the mean age was 55.3 (11.7) years. Hypertension and diabetes mellitus accounted for 82.6% and 10.5% of patients, respectively. Mean ABG was significantly higher in diabetics, with 11.70 (3.09) mmol/L, than in nondiabetics, with 8.67 (2.66) mmol/L. The 30-day mortality was 42.1% in total patients, 43.0% in patients without diabetes, and 34.6% in patients with diabetes. There was no significant difference between diabetics and nondiabetics in 30-day mortality. The baseline characteristics of the patients and their differences between nondiabetes and diabetes groups are shown in Table 1. A receiver operating characteristic analysis showed that the optimal cut-off value of critical hyperglycemia for 30-day mortality was 8.69 mmol/L, by which patients were divided into two groups: nonhyperglycemia (< 8.69 mmol/L) and hyperglycemia (≥ 8.69 mmol/L) groups. Kaplan–Meier survival curve analysis showed that the 30-day survival rate was significantly lower in patients with hyperglycemia than in those without hyperglycemia (*P* = 0.0017) (Fig. 2).

**Table 1:** Baseline characteristics of patients with Pontine hemorrhage.

	Total (*n* = 247)	Nondiabetes (*n* = 221)	Diabetes (*n* = 26)	*P* value
Sex (male)	172 (69.6%)	158 (71.5%)	14 (53.8%)	0.074^a^
Age (y)	55.3 (11.7)	54.8 (11.8)	58.9 (10.5)	0.093
Hypertension (yes)	204 (82.6%)	180 (81.4%)	24 (92.3%)	0.271^a^
Diabetes (yes)	26 (10.5%)	–	–	–
Body temperature (°C)	37.1 (1.1)	37.1 (1.1)	37.2 (1.4)	0.837
Heart rate (per min)	92.3 (22.5)	92.0 (22.6)	95.1 (21.4)	0.510
Abnormal respiration (yes)	52 (21.1%)	43 (19.5%)	9 (34.6%)	0.080^a^
Systolic BP (mm Hg)	178.8 (29.6)	178.7 (29.5)	179.4 (30.6)	0.915
GCS score (points)	7.0 (4.0–13.0)	7.0 (4.0–13.0)	6.5 (5.0–13.8)	0.795^a^
Pupillary light reflex (absent)	93 (37.7%)	82 (37.1%)	11 (42.3%)	0.670^a^
Hemoglobin (g/L)	145.73 (17.64)	145.45 (17.20)	148.04 (21.27)	0.481
White blood cell (×10^9^/L)	12.69 (5.19)	12.45 (5.23)	14.68 (4.46)	0.038
Platelet (×10^9^/L)	231.51 (71.14)	230.72 (70.62)	238.15 (76.58)	0.615
Serum potsium (mmol/L)	3.65 (0.50)	3.66 (0.51)	3.54 (0.34)	0.232
Serum sodium (mmol/L)	136.21 (4.67)	136.25 (4.70)	135.88 (4.49)	0.701
Alanine aminotransferase (U/L)	20.00 (12.00–29.50)	19.00 (12.00–28.00)	23.00 (15.50–37.50)	0.128^a^
Creatinine (μmol/L)	66.40 (52.00–82.45)	67.00 (53.00–83.80)	61.50 (49.00–73.75)	0.295^a^
ABG (mmol/L)	8.99 (2.86)	8.67 (2.66)	11.70 (3.09)	< 0.001
Volume (mL)	5.47 (2.25–8.89)	5.49 (2.35–9.15)	4.50 (1.32–6.90)	0.171^a^
Rostrocaudal extension (yes)	135 (54.7%)	119 (53.8%)	16 (61.5%)	0.456
Ventricular extension (yes)	77 (31.2%)	69 (31.2%)	8 (30.8%)	1.000^a^
Hydrocephalus (yes)	49 (19.8%)	43 (19.5%)	6 (23.1%)	0.611^a^
Death (yes)	104 (42.1%)	95 (43.0%)	9 (34.6%)	0.413

Data are expressed as mean (standard deviation) or median (interquartile range 25th–75th) for continuous variables, while categorical variables are expressed as percentages.

^a^Kruskal–Wallis rank test for continuous variables, Fisher exact probability test for categorical variables.

### Effects of risk factors on 30-day mortality by univariate analysis

In univariate analysis, the odds ratio (OR) values (per 1 mmol/L of increase in ABG) in total patients, nondiabetics, and diabetics were 1.401 (95% confidence interval [CI]: 1.251–1.568, *P* < 0.0001), 1.514 (95% CI: 1.321–1.734, *P* < 0.0001), and 1.527 (95% CI: 1.021–2.282, *P* = 0.0392), respectively. Moreover, OR values for hyperglycemia group were 5.233 (95% CI: 3.023–9.058, *P* < 0.0001) in total patients, 6.300 (95% CI: 3.489–11.375, *P* < 0.0001) in nondiabetics, and inf (95% CI: 0.000–inf, *P* = 0.9956) in diabetics, respectively, compared to nonhyperglycemia group. The results of univariate analysis of influencing the mortality are shown in Table 2.

**Table 2:** Effects of risk factors on 30-day mortality by univariate analysis.

	Total (*n* = 247)	Nondiabetes (*n* = 221)	Diabetes (*n* = 26)
ABG (mmol/L)^a^	1.401 (1.251–1.568) < 0.0001	1.514 (1.321–1.734) < 0.0001	1.527 (1.021–2.282) 0.0392
ABG (≥ 8.69 mmol/L)	5.233 (3.023–9.058) < 0.0001	6.300 (3.489–11.375) < 0.0001	Inf (0.000–inf) 0.9956
Sex (female)	0.639 (0.364–1.122) 0.1192	0.568 (0.308–1.045) 0.0688	1.786 (0.349–9.127) 0.4861
Age (year)^a^	0.981 (0.959–1.003) 0.0850	0.982 (0.959–1.005) 0.1186	0.978 (0.903–1.061) 0.5961
Hypertension (yes)	0.719 (0.371–1.390) 0.3265	0.750 (0.380–1.481) 0.4072	0.500 (0.028–9.077) 0.6393
Diabetes (yes)	0.702 (0.300–1.644) 0.4153	–	–
Body temperature (°C)^a^	2.244 (1.645–3.060) < 0.0001	2.255 (1.616–3.146) < 0.0001	2.167 (0.948–4.952) 0.0666
Heart rate (per min)^a^	1.043 (1.029–1.058) < 0.0001	1.044 (1.028–1.060) < 0.0001	1.048 (0.998–1.100) 0.0583
Abnormal respiration (yes)	10.495 (4.813–22.887) < 0.0001	10.373 (4.356–24.702) < 0.0001	26.250 (3.041–226.610) 0.0030
Systolic BP (mmHg)^a^	1.012 (1.003–1.021) 0.0109	1.013 (1.003–1.022) 0.0088	1.003 (0.976–1.030) 0.8416
GCS score (points)^a^	0.616 (0.547–0.694) < 0.0001	0.626 (0.555–0.706) < 0.0001	0.183 (0.031–1.063) 0.0585
Pupillary light reflex (absent)	16.098 (8.465–30.614) < 0.0001	13.892 (7.112–27.138) < 0.0001	Inf (0.000–inf) 0.9962
Hemoglobin (g/L)^a^	1.017 (1.001–1.032) 0.0323	1.024 (1.007–1.042) 0.0051	0.976 (0.936–1.017) 0.2455
White blood cell (×10^9^/L)^a^	1.180 (1.110–1.254) < 0.0001	1.183 (1.107–1.263) < 0.0001	1.334 (1.028–1.731) 0.0302
Platelet (×10^9^/L)^a^	1.003 (0.999–1.006) 0.1583	1.002 (0.998–1.005) 0.4029	1.013 (0.999–1.028) 0.0644
Serum potassium (mmol/L)^a^	1.263 (0.759–2.101) 0.3697	1.262 (0.748–2.129) 0.3831	0.914 (0.083–10.081) 0.9412
Serum sodium (mmol/L)^a^	0.978 (0.925–1.033) 0.4165	0.961 (0.906–1.019) 0.1866	1.168 (0.929–1.468) 0.1841
Alanine aminotransferase (U/L)^a^	1.002 (0.996–1.009) 0.4529	1.002 (0.995–1.008) 0.6268	1.025 (0.989–1.062) 0.1790
Creatinine (μmol/L)^a^	1.009 (1.001–1.016) 0.0280	1.007 (1.000-1.015) 0.0594	1.023 (0.986–1.061) 0.2240
Volume (mL)^a^	1.585 (1.405–1.788) < 0.0001	1.555 (1.376–1.758) < 0.0001	2.144 (1.101–4.177) 0.0249
Rostrocaudal extension (yes)	6.917 (3.843–12.448) < 0.0001	6.343 (3.459–11.631) < 0.0001	Inf (0.000–inf) 0.9954
Ventricular extension (yes)	5.153 (2.876–9.233) < 0.0001	4.629 (2.513–8.528) < 0.0001	15.000 (1.981–113.560) 0.0087
Hydrocephalus (yes)	3.294 (1.710–6.346) 0.0004	3.515 (1.734–7.126) 0.0005	2.333 (0.362–15.054) 0.3731

Data are expressed as OR (95% CI) *P* value.

^a^Unit odds ratio (OR), indicating the increase in OR for every 1-unit increase.

### Effects of ABG on 30-day mortality after adjusting for confounders

**Fig. 3:**
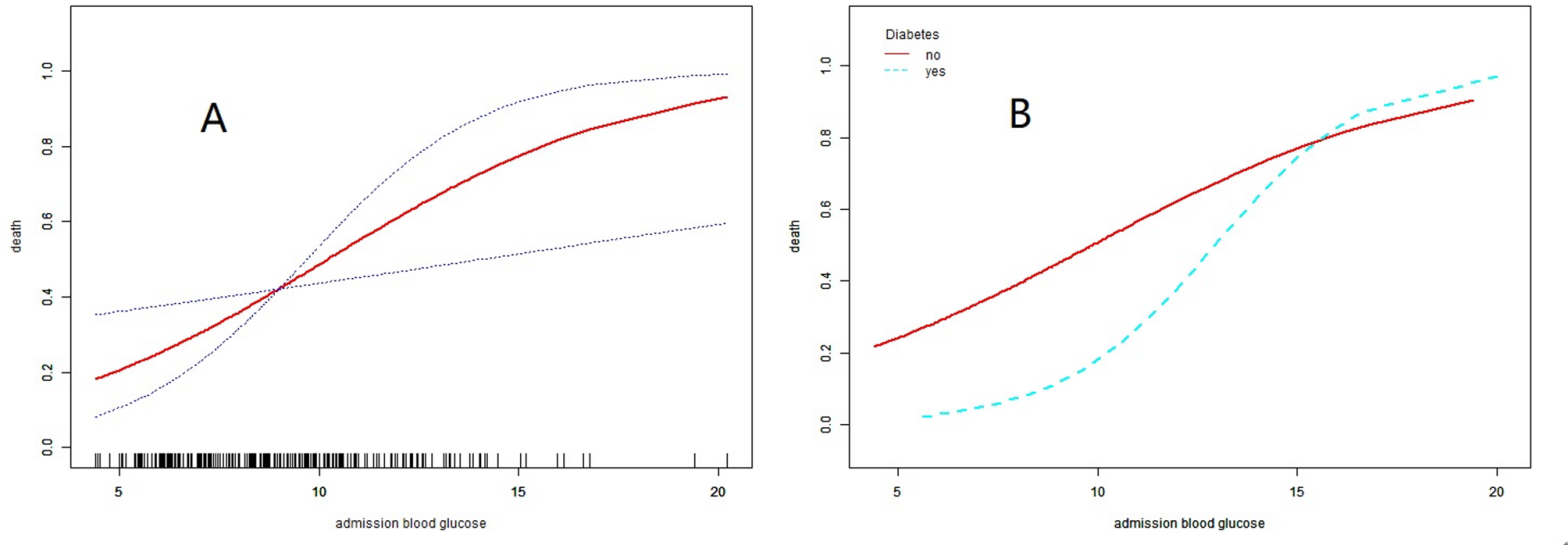
Predicted probability of outcome by admission blood glucose. **(A)** Total patients, (**B)** patients were classified as nondiabetics and diabetics. Adjusted for age, hypertension, body temperature, heart rate, systolic BP, abnormal respiration, GCS score, pupillary light reflex, hemoglobin, white blood cell, creatinine, hemorrhagic volume, rostrocaudal and intraventricular extension, hydrocephalus in **A** and **B**; and also adjusted for diabetes in **A**.

Selected confounders for multivariate analysis included age, hypertension, diabetes, body temperature, heart rate, abnormal respiration, systolic BP, pupillary light reflex, GCS score, hemoglobin, white blood cell, creatinine, hemorrhagic volume, intraventricular and rostrocaudal extension of hemorrhage, and hydrocephalus. The smoothing curve fitting indicated a linear relationship between ABG and mortality in total patients and nondiabetics after adjusting for confounders, with a trend toward increased risk of death with increase of ABG concentration (Fig. 3).

In multiple regression analysis, the OR value (per 1 mmol/L of increase in ABG) was 1.295 (95% CI: 1.065–1.575, *P* = 0.0097) in total patients after adjusting for all selected confounders (model II). The OR value for hyperglycemia group was 3.641 (95% CI: 1.579–8.394, *P* = 0.0024) after adjusting for the same confounders, compared with that of nonhyperglycemia group. Similar results were found in nondiabetics but not in diabetics, as described in Table 3. No evidence of significant interaction with diabetic status was found whether ABG was treated as a continuous variable or was classified into hyperglycemia and nonhyperglycemia groups.

**Table 3:** Effects of admission blood glucose on 30-day mortality after adjusting for confounders.

Outcome	ABG	Nonadjusted model	Adjusted model I^b^	Adjusted model II^c^
Total (*n* = 247)	mmol/L^a^	1.401 (1.251–1.568) < 0.0001	1.277 (1.097–1.485) 0.0016	1.295 (1.065–1.575) 0.0097
	< 8.69 mmol/L	1.0	1.0	1.0
	≥ 8.69 mmol/L	5.233 (3.023–9.058) < 0.0001	3.308 (1.619–6.758) 0.0010	3.641 (1.579–8.394) 0.0024
Nondiabetes (*n* = 221)	mmol/L^a^	1.514 (1.321–1.734) < 0.0001	1.317 (1.104–1.571) 0.0022	1.266 (1.036–1.547) 0.0210
	< 8.69 mmol/L	1.0	1.0	1.0
	≥ 8.69 mmol/L	6.300 (3.489–11.375) < 0.0001	3.481 (1.646–7.365) 0.0011	3.492 (1.509–8.077) 0.0035
Diabetes (*n* = 26)	mmol/L^a^	1.527 (1.021–2.282) 0.0392	Inf (0.000–inf) 0.9988	29.984 (0.000–inf) 0.9999
	< 8.69 mmol/L	1.0	1.0	1.0
	≥ 8.69 mmol/L	Inf (0.000–inf) 0.9956	Inf (0.000–inf) 0.9980	107.132 (0.000–Inf) 0.9999

Data are expressed as OR (95% CI) *P* value.

^a^Unit odds ratio (OR), indicating the increase in OR for every 1-unit increase in ABG.

^b^Adjusted for hemorrhagic volume, rostrocaudal and intraventricular extension, hydrocephalus.

^c^Adjusted for age, hypertension, body temperature, heart rate, systolic BP, abnormal respiration, GCS score, pupillary light reflex, hemoglobin, white blood cell, creatinine, hemorrhagic volume, rostrocaudal and intraventricular extension, hydrocephalus, and also adjusted for diabetes in total patients.

## Discussion

Pontine (or brainstem) hemorrhage, as a subset of acute ICH, shows more severe symptoms and higher mortality and disability rates compared to other types of ICH^1–4^. Among studies on pontine (or brainstem) hemorrhage, only a few involved the association between serum glucose level and mortality or poor outcome. Huang et al.^8^ analyzed the factors influencing 30-day mortality in a cohort of 171 patients with primary PH. They found that ABG (as a continuous variable) was significantly different between survivors and non-survivors. However, only GCS score and PH volume, but not ABG, were independent risk factors for 30-day mortality. Takeuchi et al.^9^retrospectively reviewed 212 patients with primary brainstem hemorrhage. Logistic regression analysis showed that initial blood glucose level was not an independent predictor of 3-month mortality; although univariate analysis showed a significant association between glucose levels and mortality. In contrast to findings of Huang et al. and Takeuchi et al., Fan et al.^10^ reported in their study of 225 patients with primary brainstem hemorrhage that elevated ABG, as well as increased neutrophil to lymphocyte ratio and platelet to lymphocyte ratio, was significantly associated with 90-day outcome(Glasgow outcome scale < 3). Meguro et al.^11^ also found that initial blood glucose of 180 mg/dL or higher was an independent predictor of 30-day mortality.

The present study further confirmed the results reported by Fan et al. and Meguro et al. However, the advantages of our study included a relatively large sample size and strictly performed statistical analyses to effectively control for confounders. In the present study, ABG was significantly associated with 30-day mortality in multiple regression analysis when ABG was analyzed as a continuous variable. Hyperglycemia, whose critical value was identified as 8.69 mmol/L, was also an independent risk factor for 30-day mortality. The definition of hyperglycemia, according to previous studies for ICH, was diverse, ranging from 6.0 to 10.0 mmol/L^12,13,16,17^. In the present study, the cut-off value of critical hyperglycemia falls within the ranges. Furthermore, when patients with PH were stratified into diabetic and nondiabetic subgroups, the ABG level (as a continuous variable) had a significant effect on 30-day mortality in nondiabetic patients, and hyperglycemia remained an independent risk factor for 30-day mortality. However, the phenomenon was not found in diabetic patients. Therefore, admission hyperglycemia was an independent risk factor for 30-day mortality only in nondiabetic patients. Accordingly, admission hyperglycemia was a predictor of 30-day mortality specifically in nondiabetic patients. Notably, the small sample size of diabetic patients (*n* = 26) in this study limits the generalizability of these findings to this subgroup. Therefore, the relationship between ABG and early mortality in diabetic patients with PH warrants further investigation.

Several explanations may account for the elevation of blood glucose after ICH. First, hyperglycemia is likely to be a stress response induced by ICH^17–19^which could be indicated by initial stroke severity. In our study, hyperglycemia was significantly associated with variables signifying the stroke severity, including not only larger hemorrhagic volume, but also the presence of rostrocaudal or ventricular extension and most acute clinical indicators (Table 1). These all suggest that hyperglycemia is a stress reaction to severe hemorrhage. Once the stress is initiated, neuroendocrine axes such as the locus coeruleus-sympathetic-adrenal medullary, the hypothalamic-pituitary-adrenal cortex, and hypothalamus-sympathetic-liver axis are activated, which cause excessive release of stress hormones such as catecholamines, glucagon, and cortisol^20–23^. The combined action of these stress hormones results in enhancement or acceleration of glycogenolysis, gluconeogenesis, lipolysis, and proteolysis, all finally promoting production of glucose. Second, hyperglycemia is likely to result from injury or irritation of the neuroendocrine axes caused by PH. Severe hemorrhage not only results in direct physical trauma, but is also accompanied by mass and compression effect, intraventricular extension, deformation of brainstem, constriction or occlusion of ambient cistern, elevation of intracranial pressure, and obstructive hydrocephalus, all of which may impair central glucose regulation. Meanwhile, primary pontine injury may initiate both local and systemic inflammatory response^24–26^ that disrupt metabolic homeostasis and further elevate blood glucose. Third, hyperglycemia is likely to result partly from pre-stroke abnormal glucose metabolism such as metabolic syndrome, prediabetes, and undiagnosed or even known diabetes. Patients with these conditions are more likely to have insulin resistance and impaired glucose metabolism, which may be exacerbated by the acute ICH. Furthermore, such individuals often present with vascular abnormalities, including cerebral vascular sclerosis or microangioma, which may increase their vulnerability to hemorrhage^27–29^.

The pathophysiological mechanism underlying the deteriorative effects of hyperglycemia on early mortality in PH remains to be fully elucidated. Numerous studies have identified hyperglycemia as a marker of acute stress in response to initial stroke severity, demonstrating its significant association with mortality and poor outcomes, thereby supporting its role as a prognostic predictor^5,11,16^. In our study, however, the impact of ABG on 30-day mortality persisted significantly even after adjusting for all selected confounders in Model II (including radiological and clinical variables). Although the OR values were attenuated compared to the unadjusted model or Model I (adjusted solely for radiological variables), the retained statistical significance suggests that hyperglycemia is not merely an epiphenomenon of stress response but likely exerts independent pathophysiological effects. Animal and clinical studies showed that hyperglycemia resulted in neuronal apoptosis, generation of superoxide and inflammatory cytokines, accumulation of lactate, intracellular acidosis, elevation of cytosolic free calcium, disruption of the blood–brain barrier, cerebral edema, and expansion of early hematoma^29–33^. The synergistic effects of these mechanisms result in not only direct neurotoxic effects but also aggravation of peri-ICH foci and systemic inflammatory responses^26,34,35^, all finally contributing to deterioration of the condition and unfavorable outcomes.

There are several limitations in this study. First, this study was conducted in two hospitals, and the results of the analysis may not be generally applicable. Second, because magnetic resonance imaging or angiography was not performed for all patients in critical conditions on admission, not all patients with hemorrhage secondary to aneurysm or cerebrovascular malformation were able to be ruled out. Third, this study was a retrospective and observational analysis. Therefore, a prospective study should be performed to confirm our findings.

## Conclusions

In the present study, we confirmed the significant association of ABG with 30-day mortality after PH. Admission hyperglycemia is an independent predictor for 30-day mortality. The results were found in patients without diabetes, but not in patients with diabetes. Further studies are needed to elucidate the pathophysiological mechanisms underlying the association between hyperglycemia and early mortality, and to determine whether aggressive interventions—such as targeting hemorrhage-induced brain tissue damage or actively lowering blood glucose—can ultimately reduce poor outcomes.

## Data Availability

The datasets used and/or analyzed during the current study are available from the corresponding author on reasonable request.

## Abbreviations

PH: Pontine hemorrhage
ICH: Intracerebral hemorrhage
ABG: Admission blood glucose
OR: Odds ratio
95% CI: 95% confidence interval
BP: Blood pressure
GCS: Glasgow Coma Scale
